# Bovine milk-derived exosomes enhance goblet cell activity and prevent the development of experimental necrotizing enterocolitis

**DOI:** 10.1371/journal.pone.0211431

**Published:** 2019-01-30

**Authors:** Bo Li, Alison Hock, Richard Y. Wu, Adam Minich, Steven R. Botts, Carol Lee, Lina Antounians, Hiromu Miyake, Yuhki Koike, Yong Chen, Augusto Zani, Philip M. Sherman, Agostino Pierro

**Affiliations:** 1 Translational Medicine Program, The Hospital for Sick Children, Toronto, Ontario, Canada; 2 Division of General and Thoracic Surgery, The Hospital for Sick Children, University of Toronto, Toronto, Ontario, Canada; 3 Cell Biology Program, The Hospital for Sick Children, Toronto, Ontario, Canada; 4 Developmental and Stem Cell Biology Program, The Hospital for Sick Children, Toronto, Ontario, Canada; 5 Division of Gastroenterology, Hepatology and Nutrition, The Hospital for Sick Children, University of Toronto, Toronto, Ontario, Canada; Universita degli Studi di Torino, ITALY

## Abstract

Necrotizing enterocolitis (NEC) is characterized by intestinal injury and impaired mucin synthesis. We recently showed that breast milk exosomes from rodents promote intestinal cell viability, epithelial proliferation, and stem cell activity, but whether they also affect mucus production is unknown. Therefore, the aim of this study was to investigate the effects of bovine milk-derived exosomes on goblet cell expression in experimental NEC and delineate potential underlying mechanisms of action. Exosomes were isolated from bovine milk by ultracentrifugation and confirmed by Nanoparticle Tracking Analysis and through the detection of exosome membrane markers. To study the effect on mucin production, human colonic LS174T cells were cultured and exposed to exosomes. Compared to control, exosomes promoted goblet cell expression, as demonstrated by increased mucin production and relative expression levels of goblet cell expression markers trefoil factor 3 (TFF3) and mucin 2 (MUC2). In addition, exosome treatment enhanced the expression of *glucose-regulated protein 94 (GRP94)*, the most abundant intraluminal endoplasmic reticulum (ER) chaperone protein that aids in protein synthesis. Furthermore, experimental NEC was induced in mouse pups by hyperosmolar formula feeding, lipopolysaccharide administration and hypoxia exposure on postnatal days 5–9. Milk exosomes were given with each gavage feed. NEC was associated with ileal morphological injury and reduction in MUC2+ goblet cells and GRP94+ cells per villus. Exosome administration to NEC pups prevented these changes. This research highlights the potential novel application of milk-derived exosomes in preventing the development of NEC in high-risk infants when breast milk is not available.

## Introduction

Necrotizing enterocolitis (NEC) is a devastating intestinal disease in premature and very low birth weight infants with a mortality rate of up to 50% [1]. This gastrointestinal disease ranges in severity, from ileal and/or colonic inflammation to intestinal perforation, extensive necrosis, multiple organ failure, and death.

Exosomes are cell-derived vesicles that range in diameter from ~50 to 150 nm and are formed when multi-vesicular endosomes fuse with the plasma membrane and release their intraluminal vesicles by exocytosis as exosomes [2, 3]. Exosomal phospholipid membranes encapsulate proteins, microRNA, and messenger RNA, and provide stability to these contents by protecting them from freeze-thaw cycles, ribonuclease digestion and gastric acidity [4]. These vesicles are involved in immune responses, cell adhesion, waste management, protection against stress, and inflammation, with their primary role being intercellular signaling and communication [2].

It is well known that breastfeeding protects against the development of NEC [5, 6]; however, the mechanism(s) of action mediating this protective effect remain unknown. Exosomes are released by the majority of body tissues and are therefore present in most body fluids, including breast milk [7]. Early milk such as the colostrum is a richer source of exosomes than mature milk, which contains a lower concentration of these vesicles [7]. Interestingly the onset of NEC is at 2–3 weeks of age, which may correspond with when the effects of colostrum are no longer as pronounced. We have recently shown that exosomes derived from rat milk promote intestinal epithelial cell viability, enhance cell proliferation, and stimulate intestinal stem cell activity under normal conditions [8]. However, according to our knowledge, the effect of milk-derived exosomes on the prevention of NEC has not been evaluated.

An invasion of pathogenic bacteria and an immature intestinal barrier are crucial factors that contribute to the pathogenesis of NEC [9]. Several studies have identified effective therapeutic targets to restore the gut barrier in experimental NEC [10–12]. Goblet cells are responsible for the production of mucin glycoproteins, which form a protective mucus barrier against bacterial penetration [13]. Unsurprisingly, mucin production is reduced in the ileum during NEC, making it a promising target for therapeutic intervention [14, 15].

The role of the endoplasmic reticulum (ER) is to maintain polypeptide folding and functional protein synthesis as part of a highly-regulated process facilitated by ER chaperone proteins, including GRP94, the most abundant protein in the ER lumen [16]. During episodes of ER stress, protein folding is impaired, leading to the accumulation of unfolded proteins and decreased synthesis of functional proteins. Mucins are large proteins, which are particularly susceptible to misfolding from ER stress [17, 18]. Previous literature has corroborated the link between GRP94 impairment and reduced maintenance of both goblet cells and the gut barrier [19], highlighting the potential relevance of this chaperone protein in goblet cell dysfunction and mucus loss in NEC.

To expand on our previous findings, the aims of the present study were to provide a proof-of-concept and investigate the effects of bovine milk-derived exosomes as a prevention strategy for NEC, and uncover the potential underlying mechanisms of action especially with regards to goblet cell number and expression.

## Materials and methods

### Exosome isolation

**Fig 1:**
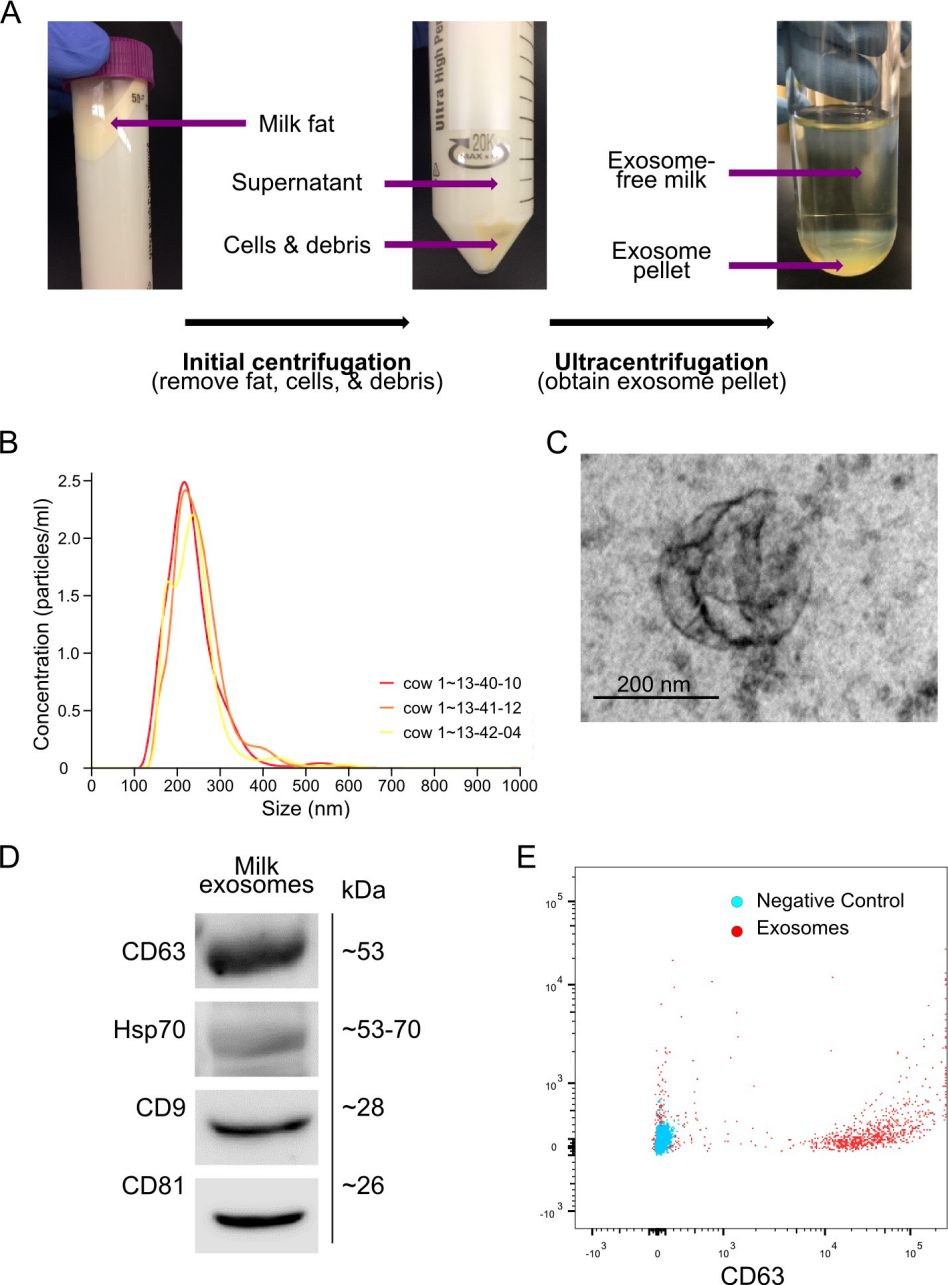
Bovine milk-derived exosome isolation, confirmation and characterization. (A) Visualization of the separated bovine milk layers following the clearing centrifugation and ultracentrifugation steps involved in the exosome isolation procedure. (B) Quantification of the concentrations of various-sized particles in the isolated exosome mixture obtained through Nanoparticle Tracking Analysis. The presence of exosomes was confirmed via the observed peak around 200 nm. (C) TEM depicts exosome morphology and the presence of exosomes as conjugates. (D) Immunoblot bands demonstrating the presence of established exosome membrane markers CD63, Hsp70, CD9 and CD81 (ExoAb Antibody Kit, SBI System Biosciences, Palo Alto, CA) in the isolated exosome mixture to confirm the successful isolation of exosomes. (E) Exosomes were sorted via flow cytometry using antibodies to CD63. Sorted positive exosomes and negative control for CD63 are shown. Experiments were independently repeated 3 times.

Bovine milk exosomes were isolated from 5–6 litres of unpasteurized fresh bovine milk obtained from a single udder of a single cow at one time from a private farm (Ontario, Canada), using ultracentrifugation, and a commonly reported method of extraction (**Fig 1A)**. All centrifugations were performed at 4°C. Initially, milk was centrifuged at 2,000 x g for 10 minutes to remove the upper layer of fat, and again at 12,000 x g for 40 minutes to eliminate deposited cells and debris. This supernatant, now devoid of fat, cells and debris, was stored at -80°C until ready for use. It has been reported that these initial centrifugation steps should be performed prior to freezing milk for exosome isolation, as it reduces the number of apoptotic bodies contaminating the sample [20]. After the milk was thawed, it went through two brief centrifugations to remove debris remaining from the freeze/thaw step: 1,200 x g for 10 minutes and 300 x g for 10 minutes. Supernatant was then passed through a 0.2μm filter for sterilization by eliminating larger particles, including bacteria. The exosome pellet was obtained by ultracentrifugation at 100,000 x g for 2 hours at 4°C using a Beckman Coulter L-90K ultracentrifuge. Following removal of the exosome-free supernatant, the pellet was re-suspended in a volume of phosphate buffered saline (PBS) equal to one fifth of the volume of milk placed in the ultracentrifuge and then stored at -20°C until use in the experiment. To label and track exosome components, ExoGlow NTA Fluorescent Labeling Kit (SBI System Biosciences, Palo Alto, CA) was used according to the manufacturer’s instructions.

### Exosome characterization

As previously reported [8], Nanoparticle Tracking Analysis was used to visualize and quantify exosomes. For Transmission electron microscopy (TEM), exosomes were sedimented by ultracentrifugation (200,000 g for 20 h in Beckman TL-100) from the appropriate sucrose gradient fractions and fixed in 3% (w/v) glutaraldehyde and 2% paraformaldehyde in cacodylate buffer, pH 7.3. The fixed exosomes were then applied to a continuous carbon grid and negatively stained with 2% uranyl acetate. The samples were examined with a Philips CM10 electron microscope.

Protein quantification of the exosomal suspension was made to ensure that there was no contamination in the purified exosome fraction by excluding the presence of detectable protein or antibodies. Encapsulating protein levels were used to quantify exosomes. Exosomal membrane protein markers were assessed by western blotting using ExoAb Antibody Kit (SBI System Biosciences, Palo Alto, CA) according to the manufacturer’s protocol. Immuno-positive bands were detected using an ECL Plus kit (Invitrogen).

Flow cytometry was performed to confirm the presence of exosomal surface makers. Briefly, exosomes were incubated with CD63 antibody at 4°C overnight, and were then incubated with FITC secondary antibody for 2 hours at room temperature, washed and analyzed by flow cytometry on a Beckman Coulter Gallios flow cytometer. Data was analyzed using FlowJo software. Exosomes not incubated with antibodies used as negative control.

### Cell culture

LS174T human colonic cells have been shown to exhibit goblet cell-like properties and have been identified as the most appropriate cell line to study mucin expression [21]. LS174T cells were obtained from Dr. Philip Sherman lab (The Hospital for Sick Children) and cultured in DMEM medium supplemented with 15% fetal bovine serum (ThermoFisher, Waltham, MA), and grown at 37°C in a humidified incubator with 5% CO_2_. Cells were treated with either 0.1 μg/μl of exosomes or a control solution (PBS) for 24 hours. Periodic Acid Schiff and Alcian blue (ScyTek, Logan, UT) stains were used to visualize mucin production, as previously reported [22].

### NEC induction

All animal experiments received ethical approval by the Animal Care Committee of The Hospital for Sick Children (no.32238) and were performed in accordance with its guidelines and regulations. C57BL/6 mice were randomly assigned to one of three groups: (i) control breastfed (control; n = 9), (ii) NEC induction (NEC; n = 9), or (iii) NEC induction plus exosome administration via gavage to mimic the normal route of breastmilk or nutrient administration in neonates. (NEC+Exosomes; n = 9). NEC was induced using a well-established protocol consisting of three stresses between postnatal days 5 and 9. NEC mice underwent 10 minutes of hypoxia (5% O_2_) prior to each feed, gavage feeding of hyperosmolar formula (three times per day), and lipopolysaccharide injection (4mg/kg) on postnatal days 6 and 7 [23, 24]. In the exosome-administered group, exosomes isolated from bovine milk as described above, were added to formula at a concentration of 1μg/μl, for each gavage feed. This concentration was chosen to equal to the amount of exosomes present in breast milk [8] and to ensure that the control group and the exosome-administered group received an equal quantity of exosomes. Several litters were used to eliminate potential litter effects as a confounding variable. Animals were monitored were treated according to the animal care protocols by the Lab Animal Services at the Hospital for Sick Children. Animals were sacrificed on postnatal day 9 by cervical dislocation and the distal ileum harvested for further analysis.

### Intestinal injury assessment

To assess tissue injury, intestinal morphology was assessed by four blinded investigators, according to a histopathology scoring system established for NEC [25, 26]. Scores of 2 or more are considered indicative of experimental NEC. Myeloperoxidase (MPO) concentration was determined using a Coulometric Activity Assay Kit (Sigma Aldrich, St. Louis, MO), as describe previously [22, 27].

### Gene expression

RNA was isolated from tissue culture cells and distal ileum using TRIzol (Invitrogen, Carlsbad, CA), according to the manufacturer’s instructions. Reverse transcription was performed on 1μg of RNA using qScript cDNA Supermix (Quanta Biosciences, Gaithersburg, MD). SYBR green-based qRT-PCR was carried out with advanced qPCR Supermix (Wisent Inc., Quebec, Canada), with the primers, *TFF3* forward CCAAGGACAGGGTGGACTG; reverse AAGGTGCATTCTGCTTCCTG, *Muc2* forward TGGGTGTCCTCGTCTCCTACA; reverse TGTTGCCAAACCGGTGGTA, *GRP94* forward AAGAATGAAGGAAAAACAGGACAAAA; reverse CAAATGGAGAAGATTCCGGC, and *Glyceraldehyde 3-phosphate dehydrogenase (GAPDH)* forward GGGGAAGGTGAAGGTCGGAG; reverse CCTGGAGATGGTGATGGGA. Data were analyzed using CFX Manager 3.1 (Biorad, Hercules, CA). Results were from three independent experiments each performed in triplicate. Expression levels were calculated by the ΔΔCt method and normalized to the reference housekeeping gene *GAPDH*.

### Immunofluorescence

Immunofluorescence staining for MPO (R&D systems, Minneapolis, MN), Muc2 (Novus Biologicals, Littleton, CO) and GRP94 (ThermoFisher, Waltham, MA) was performed to assess inflammation, goblet cell mucin production and endoplasmic reticulum function, respectively. After blocking of non-specific binding, cultured cells and tissue sections were incubated with primary antibodies (1:500 dilution) overnight at 4°C, and with fluorescent secondary antibodies (1:1000) for two hours at room temperature. DAPI (Vector Laboratories, Burlington, ON) was added for visualization of cell nuclei. Positively stained cells were counted in five contiguous villi for each coded tissue section.

### Statistical analyses

Results are presented as means ± SD, as the data were normally distributed. Data were compared using one-way ANOVA with Bonferroni correction. p<0.05 was considered significant.

## Results

### Confirmation and characterization of bovine milk-derived exosomes

The presence of exosomes was confirmed using Nanoparticle Tracking Analysis as peak around 200 nm **(Fig 1B).** Under TEM analysis of the exosome nanoparticles, exosomes appeared as conjugates **(Fig 1C)**. The presence of exosomes was also confirmed by immunoblotting for exosomal membrane markers CD63, Hsp70, CD9 and CD81 **(Fig 1D)**. Characterization of exosomes was further confirmed by flow cytometry and the presence of exosomal surface marker CD63 **(Fig 1E)**. Purified bovine milk exosomes were used in the following experiments.

### Milk exosomes protect against NEC development

**Fig 2:**
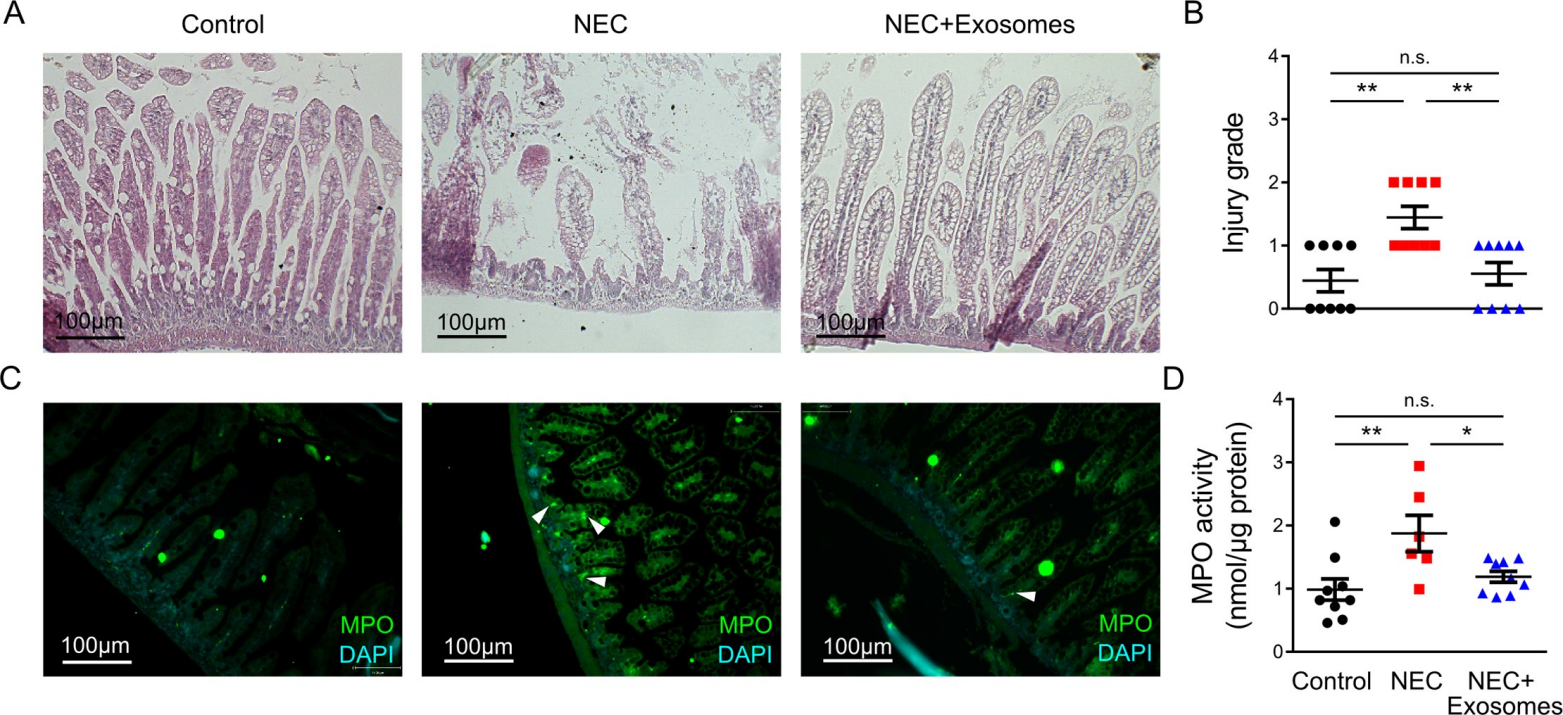
Milk exosomes protect against NEC development. (A) Representative H&E-stained histomicrographs of the terminal ileum for the three experimental groups, control, NEC, and exosome-treated NEC mouse pups. (B) Injury grades of the terminal ileum for the three experimental groups. (C) Representative MPO staining of the terminal ileum for all groups (white arrows show positive staining). (D) MPO activity for the indicated groups. Experiments were independently repeated 3 times with a total of nine mice per group. Data are presented as means ± SD. *p < 0.05; **p < 0.01, using one-way ANOVA with post-hoc tests.

In the mouse NEC model, NEC induced alterations in ileal morphology were avoided by the concomitant administration of bovine milk-derived exosomes (**Fig 2A and 2B**). In addition, NEC increased intestinal mucosal inflammation measured by MPO expression demonstrated by immune-staining and western blot, while the exosome-treated NEC group had a significant reduction in MPO expression (**Fig 2C and 2D**). These results demonstrate that milk-derived exosomes protect against NEC-induced intestinal injury.

### Milk-derived exosomes increase goblet cell expression *in vivo*

**Fig 3:**
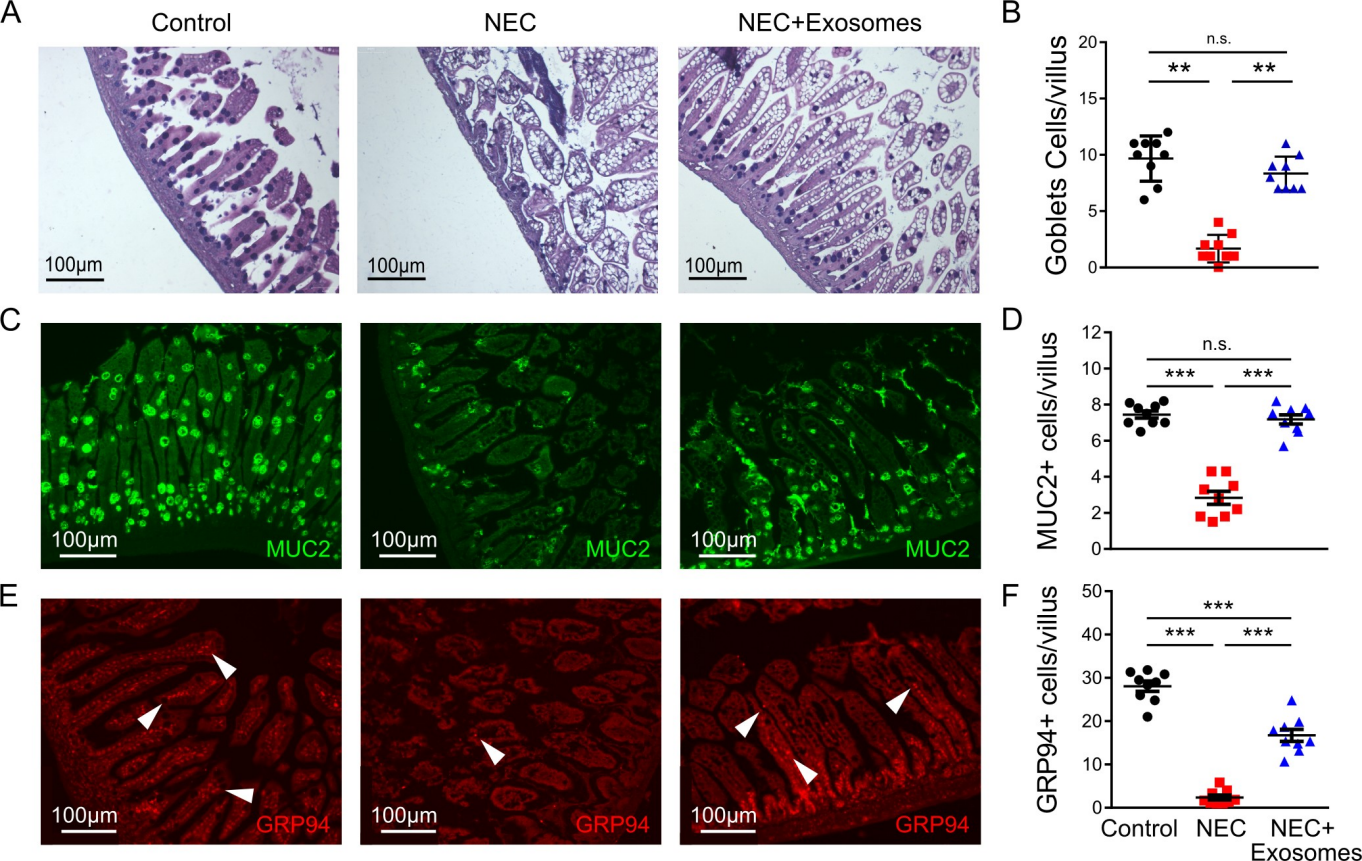
Milk-derived exosomes promote goblet cell expression *in vivo*. (A, B) Representative PAS *stained* histomicrographs and corresponding quantification of numbers of goblets cell per villus in the terminal ileum for the three experimental groups, control, NEC, and exosome-treated NEC mouse pups. (C, D) Representative micrographs for MUC2 staining and corresponding quantification of MUC2+ goblet cells per villus for control, NEC, and exosome-treated NEC mouse pups. (E-F) Representative micrographs for GRP94 staining and quantification of GRP94+ cells per villus in each experimental group. Samples were taken from the terminal ileum of each group. Experiments were independently repeated 3 times with a total of nine mice per group. Data are presented as means ± SD. ***p < 0.001, using one-way ANOVA with post-hoc tests.

Goblet cells per villus were significantly reduced during NEC compared to control. However, following administration of milk-derived exosomes in the NEC group, the number of goblet cells increased (**Fig 3A and 3B**). Similarly, the number of Muc2 positive cells per villus were decreased in NEC and rescued by exosomes treatment (**Fig 3C and 3D**). We then assessed GRP94 levels in experimental groups, as this ER chaperone protein has a vital role in goblet cell maintenance [8]. A significant reduction in GRP94-positive cells and MUC-2 positive cells per villus was observed in the ileum of mice in the NEC group, compared to control. However, the number of GRP94-positive cells were restored back to close to control levels following administration of exosomes (**Fig 3E and 3F**). Of note, administration of milk-derived exosomes to the breastfed control mice did not show significant improvement to the MUC2 or GRP94 expression levels. These results indicated that milk-derived exosomes stimulate MUC2 and GRP94 expression *in vivo* in the NEC injury model.

### Milk-derived exosomes promote goblet cell expression *in vitro*

**Fig 4:**
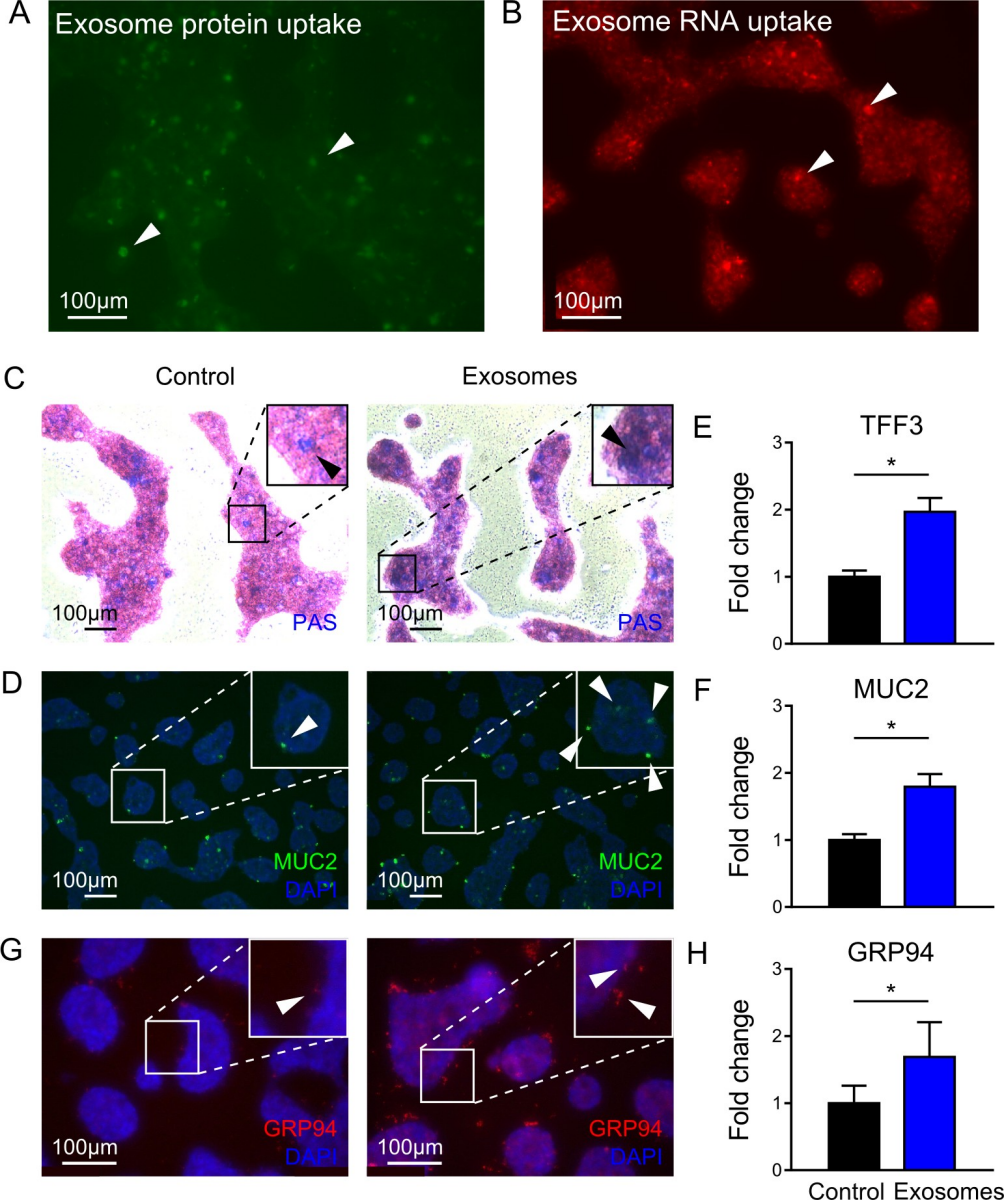
Milk-derived exosomes promote goblet cell expression *in vitro*. Exosomal protein (A) and RNA (B) content were taken up by LS174T cells. (C) Blue Periodic Acid Schiff (PAS) staining measuring the mucin production in LS174T control and exosome-treated cells (black arrows show positive stain). (D) Representative micrographs for MUC2 immunofluorescent staining of treatment and control groups (white arrows show positive stain). (E, F) Relative gene expression of goblet cell expression markers *TFF3*, and *MUC2* in both groups. (G) Representative immunofluorescent micrographs for GRP94 and (H) *GRP94* gene expression in control and exosome-treated cells. Experiments were independently repeated 3 times. Data are presented as means ± SD. *p < 0.05; using one-way ANOVA with post-hoc tests.

To confirm exosome uptake by intestinal epithelial cells, exosomes protein and RNA components were labeled and co-cultured with the human colonic goblet LS174T cells. After 4 hours, both exosomal protein and RNA content were observed within LS174T cells (**Fig 4A and 4B**). To confirm the direct impact on goblet cells, administration of milk-derived exosomes to the goblet cell line LS174T was associated with elevated levels of mucin production (blue staining, **Fig 4C**), and MUC2 expression (**Fig 4D**). Exosome treatment was also associated with increased goblet cell-associated gene expression of *TFF3* (**Fig 4E**) and *MUC2* (**Fig 4F**). Similar to *in vivo* experiments, GRP94 protein and gene expression levels were greater following exosome administration (**Fig 4G and 4H**). These *in vitro* results suggest that milk-derived exosomes direct increases MUC2 and GRP94 expression.

## Discussion

We have established an effective method for extracting and characterizing exosomes from milk. We have shown that exosomes promote intestinal epithelial cell viability, enhance proliferation, and stimulate intestinal stem cell activity under healthy conditions [8]. We have previously demonstrated that exosome-free supernatant after ultracentrifugation does not convey a protective effect, hence confirming a principal role for the exosomes in mediating the above positive effects [8]. In the present study, we extended our initial investigations by studying the effects of milk-derived exosomes administration during the induction of intestinal injury, such as NEC. We demonstrated that fortification of formula with bovine milk-derived exosomes counteracts the intestinal damage associated with experimental NEC by preventing the progression of intestinal injury and increasing goblet cell and ER functions.

Goblet cells produce mucins, which constitute the mucus layer overlying the gut surface epithelium and play an essential role in the protection of the gastrointestinal tract from injury [13]. MUC2 is the principal gel-forming mucin in the small intestine responsible for construction of the mucus barrier [13]. MUC2 is reduced in NEC-injured ileum, indicating a role for MUC2 in NEC development [14]. However, it is still not clear whether MUC2 protein depletion during inflammation may be due to functional expulsion of the mucins or to a loss of goblet cell function. Nevertheless, restoring the capacity for mucin production in goblet cells can be a novel target for NEC therapy. Indeed, we have demonstrated in this study that bovine milk-derived exosomes exert their beneficial effects on NEC prevention in experimental mice by improving goblet cell expression and mucin production. These findings are in accordance with previous studies outlining the protective effects of dietary feeding of colostrum [28] and milk oligosaccharides [29] during NEC. It has been reported that in the inflamed intestine, depletion of mucin production from goblet cells occurs prior to epithelial cell damage, inflammation and elevation of MPO [30]. In this study we have demonstrated that milk-derived exosomes reduce the expression of MPO in experimental NEC. Hence, we propose that the beneficial anti-inflammatory effect of exosomes is related to the restoration of mucin production. However, we cannot rule out the possibility of milk-derived exosomes directly modifying the host epithelial immune response.

Normal endoplasmic reticulum (ER) function is crucial for maintaining proper protein folding to synthesize functional proteins. ER stress is tightly linked to goblet cell dysfunction and altered mucin production in intestinal injury induced by the stress of maternal separation [17], inflammatory bowel diseases, and NEC [31, 32]. Many enzymes and chaperone proteins present in the ER aid in ER function; however, GRP94 is the most abundant protein within the ER lumen and has a critical link to optimal goblet cell and gut barrier function [19]. These observations are in line with the current study where we demonstrated that GRP94 expression was reduced in NEC, and that this loss can be rescued via the administration of exosomes. This beneficial effect was accompanied by the restoration of normal MUC2 levels following NEC, suggesting that exosomes could directly promote the proper folding of critical mucin proteins disrupted by NEC-induced ER stress. However, GRP94 is also essential for the folding of co-receptors critical for Wnt signaling [19], which promotes intestinal stem cell and epithelial proliferation [33]. We previously reported that breast milk-derived exosome administration stimulates intestinal stem cells and proliferation in an intestinal cell line. Thus, it is possible that exosomes restore goblet cells and MUC2 production through enhanced intestinal cell proliferation and differentiation.

Bovine milk was used as the source of milk-derived exosomes as it provides a simple model for proof-of-concept. In this study, all bovine milk was obtained from a single udder of a single animal source. However, the time of lactation in which the milk samples were obtained are not known. As recently reported by Pipino C et al, bovine milk is characterized by the presence of different types of cells [34]. Future studies are needed to define the cell origin of the milk-derived exosomes.

In the future, we aim to investigate the role of human breast milk derived exosomes to avoid potential cross-species activity. In addition, we aim to investigate whether milk exosomes induce regeneration of damaged intestinal epithelium and represent a novel treatment strategy in neonates already affected by NEC.

In summary, administration of exosomes isolated from bovine milk prevented experimental NEC-induced intestinal injury by increasing goblet cell production and ER functioning. Milk-derived exosomes present a possible preventive strategy for human infants at risk of developing NEC.
